# *GPX3* methylation in bone marrow predicts adverse prognosis and leukemia transformation in myelodysplastic syndrome

**DOI:** 10.1002/cam4.984

**Published:** 2016-11-28

**Authors:** Jing‐Dong Zhou, Jiang Lin, Ting‐Juan Zhang, Ji‐Chun Ma, Lei Yang, Xiang‐Mei Wen, Hong Guo, Jing Yang, Zhao‐Qun Deng, Jun Qian

**Affiliations:** ^1^ Department of HematologyAffiliated People's Hospital of Jiangsu UniversityZhenjiangJiangsuChina; ^2^ Laboratory CenterAffiliated People's Hospital of Jiangsu UniversityZhenjiangJiangsuChina

**Keywords:** Disease progression, *GPX3*, MDS, methylation, prognosis

## Abstract

Epigenetic inactivation of *GPX3* has been identified in various cancers including leukemia. Moreover, aberrant DNA methylation was also found as a dominant mechanism of disease progression in myelodysplastic syndrome (MDS). This study intended to explore *GPX3* promoter methylation and its clinical relevance in 110 patients with MDS. *GPX3* methylation was examined by real‐time quantitative methylation‐specific PCR (RQ‐MSP) and bisulfite sequencing PCR (BSP). *GPX3* methylation was identified in 15% (17/110) MDS patients, and significantly higher than controls, and lower than acute myeloid leukemia (AML) patients (*P *=* *0.024 and 0.041). *GPX3* methylated patients had older age and higher frequency of *DNMT3A* mutation (*P *=* *0.015 and 0.066). Cases with *GPX3* methylation showed significantly shorter overall survival (OS) time than those with *GPX3* unmethylation analyzed with Kaplan–Meier analysis (*P *=* *0.012). Moreover, Cox regression analysis revealed that *GPX3* methylation might act as an independent prognostic indicator in MDS (HR = 1.847, *P *=* *0.072). *GPX3* methylation density was significantly increased during the progression from MDS to secondary acute myeloid leukemia (sAML) in three follow‐up paired patients. Our study concludes that *GPX3* methylation in bone marrow is associated with adverse prognosis and leukemia transformation in MDS.

## Introduction

Myelodysplastic syndrome (MDS) is a heterogeneous hematopoietic stem cells (HSCs) disorder characterized by clonal hematopoiesis with quantitatively and qualitatively abnormal myeloid differentiation, and has a high risk of transforming to acute myeloid leukemia (AML) 1. The precise diagnosis of MDS is difficult, and its classification mainly depends on the cytopenias, blast percentage, morphology, and cytogenetics in bone marrow (BM) 2. Additionally, all these characteristics combined with red blood cell (RBC) transfusion dependence and patient performance status provides powerful information in the prognosis of MDS 2. Although the definite pathogenesis of MDS is not well defined, abnormal expression of proto‐oncogenes and tumor suppressor genes (TSGs) play crucial roles in the pathogenesis of MDS 3. Moreover, promoter hypermethylation of TSGs, the common mechanisms of silencing gene expression, are regarded as the drivers of MDS pathogenesis and progression to secondary AML (sAML) 4. Therefore, identifying promising biomarkers are useful for better diagnose and prognosis, to eventually plan precise therapy for MDS patients.

Glutathione peroxidase 3 (*GPX3*), a member from glutathione peroxidase (*GPX*) family (*GPX1*‐*GPX8*) located at the 5q23, accounts for almost all of the *GPX* activity in plasma, plays a crucial role in preventing oxidative damages through reducing redundant reactive oxygen species (ROS) 5. Tumor suppressor function of *GPX3* in vivo and in vitro has been identified in a number of tumors 6, 7, 8. Additionally, reduced expression of *GPX3* caused by its promoter hypermethylation has been observed in various solid tumors 9, 10, 11, 12, 13, 14, 15, 16, 17, 18, 19, 20. Simultaneously, our previous studies also have proved the epigenetic style of *GPX3* in AML and chronic myeloid leukemia (CML), and further disclosed that hypermethylation of *GPX3* promoter was a prognostically adverse indicator in AML 21, 22, 23. Here, we reported *GPX3* promoter methylation and its clinical implication in patients with MDS.

## Materials and Methods

### Patients

This study was approved by the Institutional Review Board of the Affiliated People's Hospital of Jiangsu University. After written informed consent was obtained, BM was collected from 110 patients with a diagnosis of MDS according to the 2008 revised World Health Organization (WHO) criteria 1, 24. Risk groups were classified based on International Prognosis Scoring System (IPSS) 25. The treatment for MDS patients with lower IPSS scores (Low/Int‐1) was symptomatic and supportive treatment with/without thalidomide (100 mg/d), whereas patients with higher IPSS scores (Int‐2/High) received chemotherapy which included aclacinomycin (12 mg/m^2^/d, d1‐d4), cytarabine (10 mg/m^2^/12 h, d1‐d14), granulocyte colony‐stimulating factor (200 *μ*g/m^2^/d, d0‐d14) together with symptomatic and supportive treatment. The control group was used as reported previously 22, 23.

### RNA isolation, reverse transcription, and RQ‐PCR

BM mononuclear cells (BMMNCs) were focused in this study. Total RNA was isolated form BMMNCs and was reverse transcribed as reported in our previous report 21. *GPX3* expression was evaluated by the transcript level of *GPX3* determined by real‐time quantitative PCR (RQ‐PCR) as reported 21.

### DNA isolation, chemical modification, and RQ‐MSP

Genomic DNA was isolated from BMMNCs and modified as reported previously 22, 23. The primers used for *GPX3* methylation were reported previously 17. *GPX3* methylation was detected by real‐time quantitative methyaltion‐specific PCR (RQ‐MSP) using AceQ qPCR SYBR Green Master Mix (Vazyme Biotech Co., Piscataway, NJ). RQ‐MSP was performed as our previous study 22, 23. Both positive [bisulfite‐modified DNA from K562 cells, cultured in RPMI 1640 medium containing 10% fetal calf serum (ExCell Bio, Shanghai, China)] and negative controls (ddH_2_O) were included in each assay. The normalized ratio (N_M‐*GPX3*_) was used to assess *GPX3* methylation in each sample, and calculated using the following formula: 22, 23.

$$\begin{array}{cc} N_{M-\mathrm{GPX}3}= & {\left(E_{M-\mathrm{GPX}3}\right)}^{\Delta \text{CT M‐GPX3}(\text{control‐sample})}÷\\ & {\left(E_{\mathrm{ALU}}\right)}^{\Delta \text{CT ALU}(\text{control‐sample})} \end{array}$$

### BSP

TaKaRa Taq^™^ Hot Start Version (Tokyo, Japan) kit was applied for bisulfite sequencing PCR (BSP) reaction with primers also as reported 22, 23. BSP conditions was performed as our previous study 22, 23. BSP products cloning sequencing was performed as described previously 22, 23. Six independent clones from each specimen were sequenced (BGI Tech Solutions Co., Shanghai, China).

### HRMA and DNA sequencing

High‐resolution melting analysis (HRMA) and DNA sequencing was performed in the detection of *IDH1/2*,* U2AF1*,* SF3B1*,* DNMT3A,* and *CEBPA* mutations as reported previously 26, 27, 28, 29, 30, 31.

### Statistical analyses

SPSS 20.0 software package (SPSS, Chicago, IL) was applied to the statistical analyses. Pearson Chi square analysis/Fisher's exact test was applied to compare the differences in categorical variables, whereas continuous variables were compared by Mann Whitney U test. Survival analyses were performed by both the Kaplan–Meier and the Cox regression analyses. For all analyses, a two‐tailed *P* value less than 0.05 was considered as statistically significant.

## Results

### GPX3 methylation in MDS

**Figure 1:**
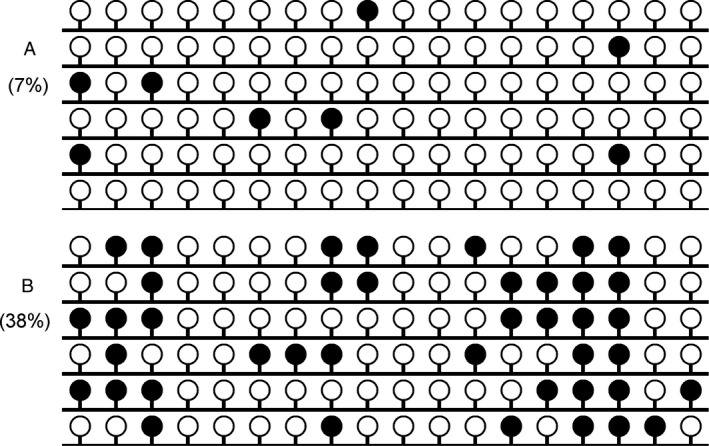
*GPX3* methylation density in one control and one myelodysplastic syndrome(MDS) patient**.** (A) Control at the cutoff point. (B) MDS patient with highest methylation level. White cycle: unmethylated CpG dinucleotide; Black cycle: methylated CpG dinucleotide.

Our previous studies have detected *GPX3* methylation in controls, AML, and CML patients, and the value of 0.184 was selected as the cutoff point to define *GPX3* methylation and unmethylation 22, 23. According to the set point, *GPX3* methylation was identified in 15% (17/110) MDS patients, and significantly higher than controls (*P *=* *0.024). Notably, the percentage of *GPX3* methylation in MDS was significantly lower than AML patients (*P *=* *0.041). Furthermore, BSP was performed in one control at the cutoff point and one MDS patient with highest methylation level showed in Figure 1.

### Association between GPX3 methylation and expression in MDS

*GPX3* transcript was further examined in 20 MDS patients and 44 controls with available samples 21. *GPX3* mRNA level was down‐regulated in MDS compared with 44 controls (median 0.042 vs. 0.348, *P *<* *0.001). Although no significantly negative correlation was observed between *GPX3* methylation and expression (*R* = −0.348, *P *=* *0.133, Spearman test, *n* = 20), patients with *GPX3* methylation (*n* = 5) showed markedly lower *GPX3* mRNA level than those without *GPX3* methylation (*n* = 15) (median 0.0002 vs. 0.0652, *P *=* *0.002).

### Association between GPX3 methylation and clinical characteristics in MDS

We further compared the clinical manifestations and laboratory features between *GPX3* unmethylated and methylated MDS patients (Table 1). No significant differences were shown in sex, peripheral blood cells, WHO classifications, cytogenetics classifications, and IPSS scores classifications (*P *>* *0.05). However, cases with *GPX3* methylation had significantly older age than those with *GPX3* unmethylation (*P *=* *0.015). Gene mutations were detected in 106 patients, and *GPX3* methylated patients tend to have higher frequency of *DNMT3A* mutation (*P *=* *0.066).

**Table 1:** Comparison of clinical manifestations and laboratory features between *GPX3* unmethylated and methylated MDS patients

Patients' parameter	Unmethylated (*n* = 93)	Methylated (*n* = 17)	Total (*n* = 110)	*P* value
Age (years)^a^	57 (14–86)	70 (38–84)	60 (14–86)	0.015
Sex (male/female)	54/39	10/7	64/46	1.000
WBC (×10^9^/L)^a^	2.7 (0.6–82.4)	3.6 (1.4–8.2)	2.75 (0.6–82.4)	0.140
HB (g/L)^a^	64 (26–128)	65 (37–118)	64 (26–128)	0.634
PLT (×10^9^/L)^a^	60 (0–1176)	59 (1–754)	60 (0–1176)	0.853
WHO				0.149
RA(RS)	11 (12%)	2 (12%)	13 (12%)	
RCMD(RS)	40 (43%)	4 (24%)	44 (40%)	
RAEB‐1	17 (18%)	5 (29%)	22 (20%)	
RAEB‐2	23 (24%)	4 (24%)	27 (25%)	
5q‐	2 (2%)	1 (6%)	3 (3%)	
MDS‐U	0 (0%)	1 (6%)	1 (1%)	
Cytogenetics				0.724
Good	62 (67%)	13 (76%)	75 (68%)	
Intermediate	16 (17%)	1 (6%)	17 (15%)	
Poor	7 (8%)	1 (6%)	8 (7%)	
No data	8 (9%)	2 (12%)	9 (8%)	
IPSS				0.310
Low	8 (9%)	3 (18%)	11 (10%)	
Int‐1	55 (59%)	6 (35%)	61 (55%)	
Int‐2	14 (15%)	4 (24%)	18 (16%)	
High	8 (9%)	2 (12%)	10 (9%)	
No data	8 (9%)	2 (12%)	10 (9%)	
Gene mutations				
*C/EBPA* (±)	2/87	1/16	3/103	0.411
*IDH1/2* (±)	3/86	1/16	4/102	0.508
*DNMT3A* (±)	1/88	2/15	3/103	0.066
*U2AF1* (±)	7/82	0/17	7/99	0.595
*SF3B1* (±)	6/83	0/17	6/100	0.586

a Median (range); WBC, white blood cells; HB, Hemoglobin; PLT, Platelet count; IPSS, International Prognostic Scoring System; WHO, World Health Organization; RA, refractory anemia; RARS, RA with ringed sideroblasts; RCMD, refractory cytopenia with multilineage dysplasia; RCMD‐RS, RCMD with ringed sideroblasts; RAEB, RA with excess of blasts. Int‐1, intermediate‐1; Int‐2: intermediate‐2.

### Association between GPX3 methylation and prognosis in MDS

**Figure 2:**
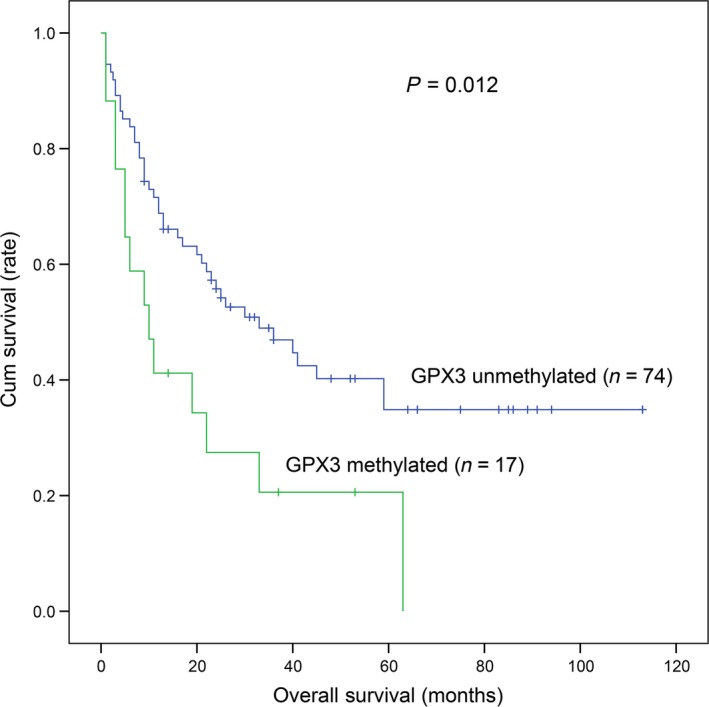
The impact of *GPX3* methylation on overall survival in MDS patients.

A total of 91 MDS patients with available survival data (range 1–113 months, median 25 months) were obtained. Kaplan–Meier analysis revealed that *GPX3* methylated patients (median: 10 months, 95% CI: 3.277–16.723 months) had markedly shorter overall survival (OS) time than *GPX3* unmethylated patients (median: 33 months, 95% CI: 15.777–50.223 months) (*P *=* *0.012, Fig. 2). To investigate whether *GPX3* methylation could act as an independent prognostic factor in MDS, Cox regression analyses (univariate and multivariate analyses) were further performed. The multivariate analysis included variables with *P *<* *0.200 in univariate analysis, and showed that *GPX3* methylation might serve as an independent risk predictor in MDS (HR = 1.847, *P *=* *0.072, Table 2).

**Table 2:** Univariate and multivariate analyses on overall survival in MDS patients

Variables	Univariate analysis		Multivariate analysis	
	Hazard ratio (95% CI)	*P* value	Hazard ratio (95% CI)	*P* value
Age	1.962 (1.128–3.412)	0.017	2.052 (1.130–3.725)	0.018
IPSS	1.547 (1.091–2.193)	0.017	1.569 (1.108–2.223)	0.011
*GPX3* methylation	2.126 (1.155–3.913)	0.015	1.847 (0.948–3.600)	0.072
*DNMT3A* mutation	3.059 (0.936–9.998)	0.064	1.838 (0.520–6.499)	0.345
*CEBPA* mutation	0.414 (0.057–3.015)	0.384	–	–
*IDH1/2* mutations	0.951 (0.296–3.054)	0.933	–	–
*U2AF1* mutation	0.780 (0.280–2.170)	0.634	–	–
*SF3B1* mutation	1.570 (0.488–5.051)	0.450	–	–

IPSS, International Prognostic Scoring System. Variables including age (≤60/>60 years old), IPSS risks (low/intermediate‐1/intermediate‐2/high), gene mutations (positive/negative), and *GPX3* methylation (methylated/unmethylated).

### Alterations in GPX3 methylation during the progression from MDS to sAML

**Figure 3:**
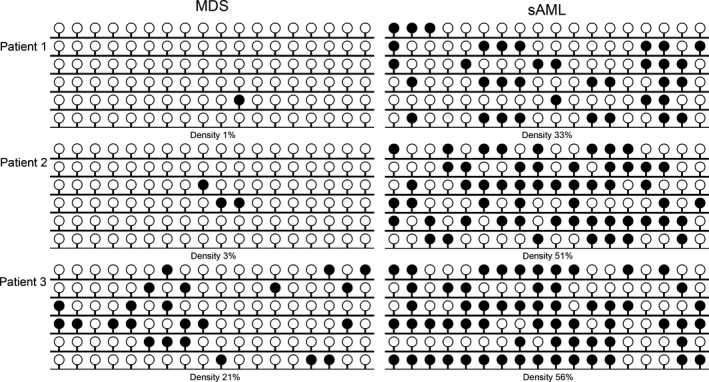
Alterations in *GPX3* methylation density during the progression from MDS to secondary AML (sAML) in three follow‐up paired patients. White cycle: unmethylated CpG dinucleotide; Black cycle: methylated CpG dinucleotide.

In order to determine whether *GPX3* methylation was involved in MDS progression to sAML, three patients with follow‐up data from MDS to sAML were further examined by BSP. As was shown in Figure 3, *GPX3* methylation density was significantly increased during the progression from MDS to sAML.

## Discussion

Tumor suppressor function of *GPX3* through the prevention of cancer initiation, promotion, and metastasis has been gradually identified in solid tumors 32. A recent study revealed that overexpression of *GPX3* suppressed tumor invasiveness, both in vivo and in vitro 8. In addition, the study also described the therapeutic value of the engineered hiPSC‐MSCs delivering *GPX3* in hepatocellular carcinoma 8. *GPX3* inhibits prostate tumorigenesis in transgenic adenocarcinoma of the mouse prostate (TRAMP) mice 33. Moreover, function role of *GPX3* has also been investigated in leukemia. Herault et al. have revealed that *GPX3* plays a vital role in the competitiveness of leukemic stem cells (LSCs) and self‐renewal activity of HSCs 34. Recently, increasing studies have demonstrated the reduced expression of *GPX3* and hypermethylation of *GPX3* promoter in various types of malignancies 9, 10, 11, 12, 13, 14, 15, 16, 17, 18, 19. Furthermore, epigenetic mechanism regulating *GPX3* expression was also identified in human cancer cells including esophageal squamous cell carcinoma (ESCC), cervical cancer (CC), gastric carcinoma (GC), and multiple myeloma (MM) 9, 13, 17, 19. With regard to leukemia, our previous studies also confirmed the epigenetic style in myeloid leukemia patients and leukemic cell lines including K562 and THP1 22, 23. These results together suggested that epigenetically silenced *GPX3* may play a vital role in both solid tumors and hematological malignancies.

In this study, *GPX3* methylation was found in 15% MDS and higher than controls but lower than AML, which indicated that *GPX3* methylation was involved in MDS pathogenesis, and might be more significant in AML. In addition, *GPX3* expression down‐regulation probably caused by promoter hypermethylation was also observed in MDS. Interestingly, Herault et al. reported that leukemia with a high frequency of LSCs expressed high levels of *GPX3* by promoter hypomethylation and low levels of ROS, which enhanced the competitiveness of LSCs 34. However, *GPX3* showed down‐regulation in clinical AML cases compared with normal CD34^+^ cells, especially in patients with favorable/intermediate karyotypes 20, 34. Taken together, these may probably be due to *GPX3* playing different roles in initial development of cancer, and *GPX3* expressed heterogeneously among different cells types like in CD34^+^ cells and mesenchymal stem cells.

DNA methylation, the most common epigenetic alteration in regulating gene expression, has been used as promising biological markers for early diagnosis, prognosis, disease progression, therapeutic stratification, and post‐therapeutic monitoring in human cancers 35. Clinical significance of *GPX3* hypermethylation has been demonstrated in a number of human cancers. The early diagnostic value of *GPX3* methylation has been revealed in ESCC 36. In addition to the diagnostic value, an increasing number of studies have also disclosed the prognostic impact of *GPX3* methylation in different types of malignancies. Peng et al. disclosed that *GPX3* methylation in GC was associated with lymph node metastasis 13. Chen et al. revealed that *GPX3* hypermethylation in head and neck cancer (HNC) correlated with chemoresistance (cisplatin‐based chemotherapy), and could serve as a potential marker predicting the prognosis of HNC patients 15. Kaiser et al. also demonstrated the negative effect of *GPX3* hypermethylation on prognosis in multiple myeloma 19. Moreover, the prognostic value of *GPX3* hypermethylation was also identified in non‐M3 AML patients 22. In this study, we also demonstrated that *GPX3* methylation might be a potential biomarker for assessing treatment outcome in MDS patients. Obviously, prospective studies are required before *GPX3* methylation can be used routinely as a novel marker for risk stratification in MDS.

To date, the underlying mechanism of leukemia transformation in MDS remains poorly defined. Genetic alterations including chromosomal abnormalities and gene mutations are considered as progression‐related drivers 37. However, these changes could not be observed among the overall process. Notably, Jiang et al. reported that epigenetic modifications of aberrant DNA methylation were a dominant mechanism in MDS progression to sAML 4. Following this study, we further determined *GPX3* methylation in follow‐up paired patients with disease progression, and demonstrated that *GPX3* methylation played a vital role in progression of MDS to sAML. However, the limited followed up paired patients calls for further studies to be able to confirm and expand our results.

Previous evidences showed that several methylation‐related gene mutations including *ASXL1*,* EZH2*,* IDH1/2*,* TET2*, and *DNMT3A* have been identified contributing to epigenetic alterations in hematological malignancies 38, 39. *DNMT3A* mutations played a crucial role in leukemogenesis and led to poor prognosis in AML and MDS 26, 40. Our study further found that *GPX3* methylation might be associated with *DNMT3A* mutation in MDS patients. Overall, these results suggested that *GPX3* methylation might be involved in MDS pathogenesis caused by *DNMT3A* mutation. Further investigations are needed to determine the specific role in MDS.

In summary, *GPX3* methylation in bone marrow is associated with adverse prognosis and leukemia transformation in MDS.

## Conflict of Interest

The authors stated that there are no conflicts of interest regarding the publication of this article.
